# A case-control study of oesophageal adenocarcinoma in women: a preventable disease

**DOI:** 10.1054/bjoc.2000.1121

**Published:** 2000-06-02

**Authors:** K K Cheng, L Sharp, P A McKinney, R F A Logan, C E D Chilvers, P Cook-Mozaffari, A Ahmed, N E Day

**Affiliations:** Department of Public Health and Epidemiology, The University of Birmingham, Edgbaston, Birmingham, B15 2TT, UK; Department of Medicine & Therapeutics, University of Aberdeen, Aberdeen, UK; Information and Statistics Division, NHS in Scotland, UK; Division of Public Health Medicine and Epidemiology, The University of Nottingham, Nottingham, UK; Division of Public Health and Primary Health Care, University of Oxford, Oxford, UK; Department of Community Medicine, University of Cambridge, Cambridge, UK

**Keywords:** oesophageal carcinoma, aetiology, obesity, fruit, breastfeeding

## Abstract

The incidence of adenocarcinoma of the oesophagus in British women is among the highest in the world. To investigate its aetiology, we conducted a multi-centre, population based case–control study in four regions in England and Scotland. We included 74 incident cases in women with histologically confirmed diagnoses of adenocarcinoma of the oesophagus, and 74 female controls matched by age and general practice. High body mass index (BMI) around the age of 20 years (highest vs lowest quartile, adjusted odds ratio (OR) = 6.04, 95% confidence interval (CI) 1.28–28.52) and low consumption of fruit (highest vs lowest quartile, adjusted OR = 0.08, 95% Cl 0.01–0.49) were associated with increases in risk. Breastfeeding by women was associated with reduced risk of their subsequently developing this cancer (ever vs never, adjusted OR = 0.41, 95% CI 0.20–0.82) and there was a significant dose–response effect with total duration of breastfeeding. The summary population attributable risk from these three factors was 96% (90% if breastfeeding is excluded). We conclude that high BMI in early adulthood and low consumption of fruit are important risk factors for adenocarcinoma of the oesophagus. Breastfeeding may confer a protective effect but this needs confirmation. This cancer is a largely preventable disease in women. © 2000 Cancer Research Campaign


*British Journal of Cancer* (2000) **83**(1), 127–132
© 2000 Cancer Research Campaign
doi: 10.1054/ bjoc.2000.1121, available online at http://www.idealibrary.com on IDEAL®

### A case–control study of oesophageal adenocarcinoma in women: a preventable disease

**KK Cheng[1], L Sharp[2], PA McKinney[3], RFA Logan[4], CED Chilvers[4], P Cook-Mozaffari[5], A Ahmed[6] and NE Day[6]**

[1]Department of Public Health and Epidemiology, The University of Birmingham, Edgbaston, Birmingham B15 2TT, UK; [2]Department of Medicine & Therapeutics, University of Aberdeen, Aberdeen, UK; [3]Information and Statistics Division, NHS in Scotland, UK; [4]Division of Public Health Medicine and Epidemiology, The University of Nottingham, Nottingham, UK; [5]Division of Public Health and Primary Health Care, University of Oxford, Oxford, UK; [6]Department of Community Medicine, University of Cambridge, Cambridge, UK

**Summary** The incidence of adenocarcinoma of the oesophagus in British women is among the highest in the world. To investigate its aetiology, we conducted a multi-centre, population based case–control study in four regions in England and Scotland. We included 74 incident cases in women with histologically confirmed diagnoses of adenocarcinoma of the oesophagus, and 74 female controls matched by age and general practice. High body mass index (BMI) around the age of 20 years (highest vs lowest quartile, adjusted odds ratio (OR) = 6.04, 95% confidence interval (CI) 1.28–28.52) and low consumption of fruit (highest vs lowest quartile, adjusted OR = 0.08, 95% CI 0.01–0.49) were associated with increases in risk. Breastfeeding by women was associated with reduced risk of their subsequently developing this cancer (ever vs never, adjusted OR = 0.41, 95% CI 0.20–0.82) and there was a significant dose–response effect with total duration of breastfeeding. The summary population attributable risk from these three factors was 96% (90% if breastfeeding is excluded). We conclude that high BMI in early adulthood and low consumption of fruit are important risk factors for adenocarcinoma of the oesophagus. Breastfeeding may confer a protective effect but this needs confirmation. This cancer is a largely preventable disease in women. © 2000 Cancer Research Campaign

**Keywords:** oesophageal carcinoma; aetiology; obesity; fruit; breastfeeding

Adenocarcinoma of the oesophagus has been increasing in incidence in most developed countries in the last two decades (Powell and McConkey, 1990; Blot et al, 1991). The incidence of this condition in British women is among the highest in the world, with half of all cases in Europe occurring in the UK (Black et al, 1997). Previous studies, which had predominantly included men, had identified obesity, diet low in fruit and vegetables, and smoking as the main risk factors (Brown et al, 1995; Vaughan et al, 1995; Gammon et al, 1997; Chow et al, 1998; Lagergren et al, 1999). Little is known about the causes of this cancer in women. Here we report a multi-centre, population-based case–control study among British women together with an estimate of the overall population attributable risk of important risk factors in a multivariate fashion.

### METHODS

This population-based case–control study was conducted in the former Regional Health Authorities (RHA) of East Anglia and Oxford, part of Trent RHA and Eastern Scotland covering the Health Boards of Highland, Grampian, Tayside, Fife, Lothian and Forth Valley. Ethical approval was given by all the local research ethics committees.

Cases comprised all women aged under 75 years of age (80 years in Trent) resident in the study areas at the time of their diagnosis with oesophageal cancer. Results on adenocarcinoma only are reported here. Cases were identified through pathology departments, treating clinicians and cancer registries and all tumours were histologically confirmed. Care was taken to exclude tumours established as arising in the cardia of the stomach but a small number of cases of those arising at the gastro-oesophageal junction may be included.

Cases were accrued over a 2-year period in each study region between 1993 and 1996. A single female control was matched to each case by age (within 5 years) and general practice. Potential controls were randomly selected using the Family Health Service Authority (FHSA) or Health Board primary care registers. Eligible controls who declined to take part were replaced.

Women were approached with consultant or General Practitioner (GP) permission and asked for a personal interview. Trained interviewers used a standard form to conduct interviews either in hospital or at home. Information was collected on sociodemographic characteristics, smoking, alcohol, tea and coffee consumption, diet, previous medical and obstetric histories, and a number of other factors, including weight, height and use of vitamin supplements. Smoking was measured in pack years and total years of smoking whilst units of alcohol were categorized by average weekly and total lifetime consumption. A dietary questionnaire was used to obtain information for recent diet (3 years prior to interview) and at age 30 years. Consumption of fresh fruit, salad and vegetables was assessed by questions on food frequency. Categories for analysis were based on quartiles of the frequency of consumption per week among all controls (including those for cases of other histological diagnoses). Only results on recent diet are presented as patterns for both early and more recent time periods were strongly correlated.

*Received 4 January 2000*
*Revised 26 January 2000*
*Accepted 26 January 2000*

*Correspondence to:* KK Cheng

**Table 1** Age and social class of cases and controls

| | No. of | | |
|---|---|---|---|
| | Controls ($n$ = 74) | Cases ($n$ = 74) | |
| Age | | | |
| < 50 | 5 | 4 | |
| 50–59 | 12 | 14 | |
| 60–69 | 26 | 29 | |
| ≥ 70 | 31 | 27 | |
| | | | OR (95% CI) |
| Social class | | | |
| I & II | 38 | 31 | 1 |
| IIIA | 6 | 7 | 1.36 (0.42–4.37) |
| IIIB | 18 | 23 | 1.69 (0.73–3.89) |
| IV & V | 9 | 12 | 1.96 (0.58–6.64) |
| Armed forces | 3 | 1 | 0.48 (0.05–4.96) |

**Table 2** Unadjusted ORs for adenocarcinoma of the oesophagus according to dietary factors

| Variable | No. of | | OR (95% CI) | *P* for trend |
|---|---|---|---|---|
| | Controls | Cases | | |
| Usual breakfast pattern | | | | |
| No breakfast | 6 | 8 | 1 | |
| Cooked breakfast | 17 | 28 | 1.09 (0.35–3.42) | |
| Other type of breakfast | 51 | 38 | 0.56 (0.18–1.70) | – |
| All vegetables* (items per week) | | | | |
| $Q_1$:0–15.37 | 14 | 18 | 1 | |
| $Q_2$:15.38–19.77 | 22 | 21 | 0.73 (0.28–1.94) | |
| $Q_3$:19.78–25.89 | 19 | 21 | 0.84 (0.31–2.33) | |
| $Q_4$:≥25.90 | 19 | 14 | 0.58 (0.22–1.55) | 0.371 |
| All salad vegetables (items per weeks) | | | | |
| $Q_1$:0–6.44 | 21 | 26 | 1 | |
| $Q_2$:6.45–11.46 | 21 | 23 | 0.93 (0.42–2.04) | |
| $Q_3$:11.47–17.11 | 15 | 19 | 1.09 (0.43–2.79) | |
| $Q_4$:≥17.12 | 17 | 6 | 0.31 (0.10–0.92) | 0.083 |
| Total fruit (items per week) | | | | |
| $Q_1$:0–12.00 | 18 | 31 | 1 | |
| $Q_2$:12.01–18.04 | 17 | 17 | 0.46 (0.16–1.31) | |
| $Q_3$:18.05–25.72 | 18 | 18 | 0.44 (0.14–1.41) | |
| $Q_4$:≥25.73 | 21 | 8 | 0.18 (0.05–0.57) | 0.003 |
| Fruit juice | | | | |
| Never | 21 | 26 | 1 | |
| <1/day | 27 | 30 | 0.88 (0.40–1.94) | |
| 1/day | 8 | 9 | 0.86 (0.27–2.69) | |
| >1/day | 18 | 9 | 0.40 (0.14–1.11) | 0.086 |
| Tea (volume per day) | | | | |
| Never/<1/day | 7 | 9 | 1 | |
| ≤6 dcl | 30 | 30 | 0.83 (0.26–2.63) | |
| 7–11 dcl | 19 | 24 | 0.95 (0.30–3.08) | |
| ≥ 12 dcl | 18 | 11 | 0.49 (0.14–1.72) | 0.309 |
| Coffee (volume per day) | | | | |
| Never/<1/day | 22 | 22 | 1 | |
| ≤3 dcl | 25 | 15 | 0.65 (0.27–1.53) | |
| 4–7 dcl | 12 | 15 | 1.62 (0.53–4.94) | |
| ≥8 dcl | 15 | 22 | 1.95 (0.68–5.57) | 0.158 |
| Preference of temperature of tea or coffee | | | | |
| Warm | 15 | 20 | 1 | |
| Hot | 42 | 42 | 0.75 (0.32–1.76) | |
| Very/burning hot | 17 | 12 | 0.51 (0.18–1.45) | 0.202 |

* Except potatoes and salad vegetables

Daily beverage consumption was estimated in decilitres for tea, coffee, other hot drinks and the temperature at the time of drinking reported as burning hot, hot and warm.

Of the 416 cases we identified as eligible, 256 (62%) cases were interviewed. Being too ill to be interviewed was the reason in the majority of those we failed to recruit. The histological diagnoses

*© 2000 Cancer Research Campaign*

**Table 3** Unadjusted ORs for adenocarcinoma of the oesophagus according to smoking and alcohol drinking habits

| Variable | No. of | | OR (95% CI) | *P* for trend |
|---|---|---|---|---|
| | Controls | Cases | | |
| Ever taken as much as one alcoholic drink per month | | | | |
| No | 14 | 22 | 1 | |
| Yes | 60 | 52 | 0.38 (0.14–1.08) | – |
| Total lifetime alcohol consumption (units) | | | | |
| Non-drinker | 14 | 22 | 1 | |
| ≤2880 | 20 | 18 | 0.42 (0.14–1.24) | |
| 2881–8312.4 | 21 | 17 | 0.32 (0.09–1.11) | |
| ≥8312.5 | 18 | 16 | 0.37 (0.11–1.25) | 0.154[a] |
| Not known | 1 | 1 | | |
| Average weekly alcohol consumption over lifetime (units/week) | | | | |
| Non-drinker | 14 | 22 | 1 | |
| <2 | 25 | 26 | 0.44 (0.15–1.29) | |
| 2–13.99 | 32 | 22 | 0.28 (0.09–0.90) | |
| ≥14 | 2 | 3 | 0.66 (0.08–4.96) | 0.074[a] |
| Not known | 1 | 1 | | |
| Smoking status | | | | |
| Never smoked | 28 | 27 | 1 | |
| Ex-smoker | 35 | 33 | 0.99 (0.53–1.87) | |
| Current smoker | 11 | 14 | 1.37 (0.51–3.70) | 0.642 |
| Total years of smoking | | | | |
| Never smoked | 28 | 27 | 1 | |
| Ex-smoker | 35 | 33 | 1.00 (0.53–1.91) | |
| ≤37.68 | 6 | 2 | 0.22 (0.02–2.00) | |
| 37.69–48.57 | 3 | 6 | 2.15 (0.38–12.06) | |
| ≥48.58 | 2 | 6 | 3.00 (0.58–15.64) | 0.166[b] |
| Pack-years | | | | |
| Never smoked | 28 | 27 | 1 | |
| Ex-smoker | 35 | 32 | 1.00 (0.53–1.91) | |
| ≤16.63 | 6 | 2 | 0.42 (0.08–2.24) | |
| 16.64–32.02 | 2 | 5 | 2.26 (0.41–12.51) | |
| ≥32.03 | 2 | 7 | 5.48 (0.63–47.93) | 0.072[a,b] |
| Not known | 1 | 1 | | |

[a]Subjects with missing values and their matched cases/controls are excluded. [b]Never and ex-smokers combined.

among cases were as follows: 159 (62.1%) had squamous cell carcinomas, 74 had adenocarcinoma (28.9%) and 23 (9.0%) were of other histologies. The response rate for first choice controls was 65%.

Data were analysed using conditional logistic regression with the use of EGRET (Statistics and Epidemiology Research Corporation, 1992) to produce odds ratios (OR), 95% confidence intervals (CI), *P*-values and deviance $\chi^2$ tests for effects. Dose–response relationships were tested for trend (Breslow and Day, 1980). Population attributable risks were estimated using the methods of Bruzzi et al (1985).

### RESULTS

The geographical distribution of the 74 cases was as follows: 31 in Eastern Scotland, 14 in East Anglia, 16 in Trent and 13 in Oxford. Table 1 shows the age and social class distribution of the cases and controls. The mean ages of cases and controls were 65.9 years and 65.3 years respectively. There was a moderate but statistically insignificant increase in risk from social classes I and II to IV and V (*P* for linear trend = 0.09 excluding four subjects and their matched cases/controls in the category ‘Armed forces’).

Table 2 shows the ORs associated with various dietary factors. Higher consumption of fruit was associated with a strong protective effect. There was also a clear linear trend (*P* = 0.003). For use of salad and fruit juice, there was a suggestion of a protective effect with high intake. The trends for both were of borderline statistical significance.

Smoking and alcohol drinking were not significantly associated with risk (Table 3), although there was a suggestion that long duration of smoking and high number of pack years may carry an increase in risk. At univariate level, alcohol drinking was associated with a statistically insignificant reduction in risk.

Body mass index (BMI) at about the age of 20 years was positively associated with risk (Table 4). Having ever breastfed carried a reduced risk and there was stronger protection with total duration of breastfeeding longer than 6 months (median value among those who had breastfed). A history of ever being diagnosed as having diabetes mellitus was associated with an elevated risk of borderline statistical significance. More cases than controls had a history of indigestion, especially of longer duration. There was some evidence of a protective effect of regular aspirin use but this did not reach statistical significance.

Table 5 shows the results of multivariate modelling. Three variables remained in the final model, namely body mass index, fruit consumption and breastfeeding. The population attributable risks associated with them were respectively 56%, 77% and 58%. The summary population attributable risk from these three factors was 96%.

*© 2000 Cancer Research Campaign*

**Table 4** Unadjusted ORs for adenocarcinoma of the oesophagus according to BMI, reproductive and medical factors

| Variable | No. of | | OR (95% CI) | *P* for trend |
|---|---|---|---|---|
| | Controls | Cases | | |
| Body mass index at 20 years of age (kg/m$^2$) | | | | |
| $Q_1$: ≤19.48 | 17 | 9 | 1 | |
| $Q_2$: 19.49–20.95 | 21 | 12 | 0.97 (0.31–3.01) | |
| $Q_3$:20.96–22.66 | 17 | 21 | 2.70 (0.84–8.73) | |
| $Q_4$:≥22.97 | 13 | 30 | 4.68 (1.53–14.35) | 0.001[a] |
| Not known | 6 | 2 | 0.27 (0.03–2.57) | |
| Number of children | | | | |
| None | 8 | 13 | 1 | |
| 1 or 2 | 40 | 31 | 0.46 (0.16–1.31) | |
| ≥3 | 26 | 29 | 0.69 (0.23–2.01) | 0.901[a] |
| Not known | 0 | 1 | | |
| Breastfeeding | | | | |
| No children/never breastfed | 18 | 34 | 1 | |
| Ever breastfed | 56 | 38 | 0.41 (0.20–0.82) | – |
| Not known | 0 | 2 | | |
| Total duration of breastfeeding | | | | |
| No children | 8 | 13 | 1 | |
| Had children but never breastfed | 10 | 21 | 0.96 (0.27–3.42) | |
| Up to 6 months | 27 | 23 | 0.42 (0.13–1.29) | |
| > 6 months | 29 | 15 | 0.31 (0.09–1.02) | 0.005[a] |
| Not known | 0 | 2 | | |
| Ever diagnosed with diabetes | | | | |
| No | 73 | 67 | 1 | |
| Yes | 1 | 7 | 7.00 (0.86–56.89) | – |
| Indigestion | | | | |
| Never suffered | 38 | 21 | 1 | |
| Indigestion for less than 2 weeks | 18 | 18 | 2.39 (0.90–6.29) | |
| Indigestion for more than 2 weeks | 18 | 35 | 4.50 (1.75–11.57) | <0.0001 |
| Ever taken aspirin daily for as long as a month | | | | |
| No | 61 | 63 | 1 | |
| Yes | 13 | 9 | 0.67 (0.27–1.63) | – |
| Not known | 0 | 2 | | |
| Ever taken vitamin/mineral supplement for 6 months or more | | | | |
| No | 38 | 48 | 1 | |
| Yes | 36 | 26 | 0.57 (0.29–1.12) | – |

[a] Subjects with missing values and their matched cases/controls are excluded.

**Table 5** Variables in the final model

| Variable | OR[a] (95% CI) | *P* for trend |
|---|---|---|
| Body mass index at 20 years of age (kg m$^{-2}$) | | |
| $Q_1$: ≤19.48 | 1 | |
| $Q_2$: 19.49–20.95 | 0.86 (0.17–4.32) | |
| $Q_3$: 20.96–22.66 | 4.90 (0.86–28.02) | |
| $Q_4$: ≥22.67 | 6.04 (1.28–28.52) | 0.002 |
| Total fruit consumption (times per week) | | |
| $Q_1$: ≤12.00 | 1 | |
| $Q_2$: 12.01–18.04 | 0.42 (0.09–2.03) | |
| $Q_3$: 18.05–25.72 | 0.37 (0.05–2.59) | |
| $Q_4$: ≥25.73 | 0.08 (0.01–0.49) | 0.002 |
| Breastfeeding | | |
| No children | 1 | |
| Had children but never breastfed | 0.66 (0.06–6.88) | |
| Up to 6 months | 0.30 (0.04–2.30) | |
| > 6 months | 0.13 (0.01–1.40) | 0.005 |

[a] Mutually adjusted and for social class and number of children.

 © 2000 Cancer Research Campaign

### DISCUSSION

In many countries, the incidence of adenocarcinoma of the oesophagus has been rising at a faster rate than almost all other cancers (Powell and McConkey, 1990; Blot et al, 1991). A number of recent studies have examined the aetiology, largely in men (Brown et al, 1995; Vaughan et al, 1995; Gammon et al, 1997; Chow et al, 1998; Lagergren et al, 1999). The significance of the present study is underlined further by the fact that the incidence of this condition is higher among British women than any other populations (Parkin et al, 1997). Although the sample size of this study was modest, we actually had included more female cases than any other study reported to date. In common with findings from previous studies in men, we found that higher BMI and having a diet low in fruit were associated with increases in risk.

The information on BMI related to young adulthood. There was a very strong trend of increasing risk with increasing BMI although most individuals in even the highest quartile would not be regarded as overweight or obese by current standards. We do not have information on recent BMI but a comparison with the general population aged 55–74 currently suggests that women in the present study had put on weight since their early adulthood. The relationship between BMI and risk of adenocarcinoma of the oesophagus is likely to be a causal one for the following reasons: (a) its strength; (b) recall bias was unlikely, the hypothesis not being well known; (c) there was a clear dose–response relationship; and (d) the association was specific to adenocarcinoma and was not found for squamous cell carcinoma (to be reported separately), suggesting that bias was unlikely. The relation, moreover, is consistent with previous reports predominantly of men (Brown et al, 1995; Vaughan et al, 1995; Chow et al, 1998; Lagergren et al, 1999). Compared with the other three studies, we, like Lagergren et al (1999) found a larger relative risk. However, one difference between Lagergren et al (1999) and the present study is that our risk estimates for BMI at the age of 20 were higher than those found by Lagergren and his colleagues, but comparable to their estimates for 20 years before interview. There is no ready explanation for the difference but possible reasons include chance and sex difference, cases in Lagergren et al (1999) being predominantly men.

Existing evidence indicates strongly that the rapid rise in incidence of adenocarcinoma of the oesophagus can be explained in part by the increase in obesity in the general population (Seidell and Flegal, 1997). However, the pathway through which obesity increases risk is unclear. One obvious possibility is that abdominal obesity predisposes individuals to reflux oesophagitis, thereby leading to Barrett’s oesophagus and adenocarcinoma. We do not have detailed information on reflux symptoms but we found that a history of indigestion was very strongly related to risk. Similar to Lagergren et al (1999), however, we found that the relationship between BMI and risk was essentially unaltered by adjusting for history of indigestion (data not shown). Further research is needed.

A history of diabetes was found to be associated with risk in univariate analyses. There was considerable confounding by obesity, as the effect was attenuated after adjusting for BMI. However, it has been reported that gastric emptying is delayed and dyspepsia is common in diabetic patients (Mearin and Malagelada, 1995).

Like previous studies (Brown et al, 1995; Zhang et al, 1997), we found that high consumption of fruit was protective. Similarly, our data suggested an increased risk with heavy smoking of long duration and reduction in risk with frequent use of salad vegetables. However, these findings were not statistically significant, possibly due to limited sample size. We did not find any increase in risk associated with alcohol consumption, although only 2/73 controls and 3/73 cases reported consumption of 14 units of alcohol or more a week. If anything, there was some suggestion of a negative association in univariate analyses, but this disappeared after adjusting for other risk factors.

The negative association with breastfeeding has not been previously described but is intriguing. It could be a chance finding, there being no a priori hypothesis. The question on breastfeeding was included to examine whether it increased the risk of squamous cell carcinoma via an effect on the nutritional balance of women. Social class or other factors we have included did not confound the effect. The finding was adjusted for number of children, but persisted if non-parous women were excluded. Though adjusted for BMI in early adulthood, it is possible that breastfeeding is inversely associated with weight gain after pregnancy (on which we have no data). Bias arising from healthy controls recalling history of breastfeeding more completely than ill cases is unlikely, as we did not find any relationship with squamous cell carcinoma. There was also a dose–response relationship between duration of breastfeeding and risk. The finding requires confirmation by further studies.

We show that high BMI and low fruit consumption accounted for 90% of the risk of the condition in this population. Against the background of the generally low fruit intake (Thompson et al, 1999) and increasing trend in the prevalence of obesity (Seidell and Flegal, 1997) in the UK, the preventability of this cancer should present an important public health opportunity.

### ACKNOWLEDGEMENTS

We would like to dedicate this paper to the memory of the late Calum Muir, who played an important role in the initiation and implementation of this study. We thank all our study coordinators and interviewers: Sandra Bonney, Gwyn Campion, Jane Davies, Gail Faulkner, Marie Fleming, Jean Fraser, Margaret Rayner, Doreen Refaat, Cherry Robertson, Jane Shearer, and Rosemary Wylie. Thanks are also due to the pathologists, clinicians and GPs who assisted us in the study, and to Roger Maric for his comments on the manuscript. Funding was provided by Chief Scientist Office, Scottish Office; the LORS in East Anglia; Special Trustees to the Nottingham University Hospitals in Trent; and the Medical Research Council in Oxford.

### REFERENCES

Black RJ, Bray F, Ferlay J and Parkin DM (1997) Cancer incidence and mortality in the European Union: cancer registry data and estimates of national data for 1990. *Eur J Cancer* **33**: 1075–1107

Blot WJ, Devesa SS, Kneller RW and Fraumeni JF Jr (1991) Rising incidence of adenocarcinoma of the esophagus and gastric cardia. *JAMA* **265**: 1287–1289

Breslow NE and Day NE (1980) *Statistical Methods in Cancer Research: Vol. 1 The Analysis of Case–control Studies*. IARC Scientific Publications, No 32. IARC: Lyon

Brown LM, Swanson CA, Gridley G, Swanson GM, Schoenberg JB and Greenberg RS (1995) Adenocarcinoma of the esophagus: role of obesity and diet. *J Natl Cancer Inst* **87**: 104–109

Bruzzi P, Green SB, Byar DP, Brinton LA and Schairer C (1985) Estimating population attributable risk for multiple risk factors using case–control data. *Am J Epidemiol* **122**: 904–914

© 2000 Cancer Research Campaign

Chow WH, Blot WJ, Vaughan TL, Risch HA, Gammon MD, Stanford JL, Dubrow R, Schoenberg JB, Mayne ST, Farrow DC, Ahsan H, West AB, Rotterdam H, Niwa S and Fraumeni JF Jr (1998) Body mass index and risk of adenocarcinoma of the esophagus and gastric cardia. *J Natl Cancer Inst* **90**: 150–155

Gammon MD, Schoenberg JB, Ahsan H, Risch HA, Vaughan TL, Chow WH, Rotterdam H, West AB, Dubrow R, Stanford JL, Mayne ST, Farrow DC, Niwa S, Blot WJ and Fraumeni JF Jr (1997) Tobacco, alcohol and socioeconomic status and adenocarcinoma of the esophagus and gastric cardia. *J Natl Cancer Inst* **89**: 1277–1284

Lagergren J, Bergstrom R and Nyren O (1999) Association between body mass and adenocarcinoma of the esophagus and gastric cardia. *Ann Intern Med* **130**: 883–890

Mearin F and Malagelada JR (1995) Gastroparesis and dyspepsia in patients with diabetes. *Eur J Gastroenterol Hepatol* **7**: 717–723

Parkin DM, Whelan SL, Ferlay J, Raymond L and Young J (1997) *Cancer Incidence in Five Continents, Vol VII*. IARC Scientific Publications No 143. IARC: Lyon

Powell J and McConkey CC (1990) Increasing incidence of adenocarcinoma of the gastric cardia and adjacent sites. *Br J Cancer* **62**: 440–443

Seidell JC and Flegal KM (1997) Assessing obesity: classification and epidemiology. *Br Med Bull* **53**: 238–252

Statistics and Epidemiology Research Corporation (1992) EGRET. SERC: Seattle.

Thompson RL, Margetts BM, Speller VM and McVey D (1999) The Health Education Authority's health and lifestyle survey 1993: who are the low fruit and vegetable consumers? *J Epidemiol Community Health* **53**: 294–299

Vaughan TL, Davis S, Kristal A and Thomas DB (1995) Obesity, alcohol, and tobacco as risk factors for cancer of the oesophagus and gastric cardia: adenocarcinoma versus squamous cell carcinoma. *Cancer Epidemiol Biomarkers Prev* **4**: 85–92

Zhang ZF, Kurtz RC, Yu GP, Sun M, Gargon N, Karpeh M Jr, Fein JS and Harlap S (1997) Adenocarcinoma of the esophagus and gastric cardia: the role of diet. *Nutr Cancer* **27**: 298–309

 *© 2000 Cancer Research Campaign*
